# Maternal serum uric acid before 24 weeks of gestation and subsequent gestational diabetes mellitus: a multicentre retrospective cohort study

**DOI:** 10.3389/fendo.2026.1896745

**Published:** 2026-07-24

**Authors:** Qinquan Cheng, Yuan Chen, Wenkai Zhang, Saijie Ma, Hua Tian, Shijie Wu, Yuan Jiang, Xiangdong Huang, Hanping Yang, Mingming Ding, Minghao Kong

**Affiliations:** 1 Department of Clinical Laboratory Medicine, Shanghai Tenth People’s Hospital, Tongji University School of Medicine, Shanghai, China; 2 Department of Obstetrics and Gynecology, The People’s Hospital of Lincang, Lincang, Yunnan, China; 3 Department of Medical Intensive Care Unit, Maternal and Child Health Hospital of Hubei Province, Tongji Medical College, Huazhong University of Science and Technology, Wuhan, Hubei, China; 4 Kidney and Metabolism Center, Shanghai Tenth People’s Hospital, Tongji University School of Medicine, Shanghai, China; 5 Department of Obstetrics, Gong’an County Hospital, Jingzhou, Hubei, China; 6 Department of Nursing, Shanghai Tenth People’s Hospital, Tongji University School of Medicine, Shanghai, China; 7 Department of Medical Intensive Care Unit, The People’s Hospital of Lincang, Lincang, Yunnan, China; 8 Department of Information Management, Shanghai Tenth People’s Hospital, Tongji University School of Medicine, Shanghai, China; 9 Department of Emergency Medicine, Shanghai Tenth People’s Hospital, Tongji University School of Medicine, Shanghai, China, China

**Keywords:** cardiometabolic risk, endocrine metabolism, gestational diabetes mellitus, hypertensive disorders of pregnancy, maternal uric acid, pregnancy, retrospective cohort

## Abstract

**Introduction:**

This multicentre retrospective cohort study examined whether maternal serum uric acid measured before 24 weeks of gestation was associated with subsequent gestational diabetes mellitus (GDM), with hypertensive disorders of pregnancy (HDP) assessed as a related cardiometabolic outcome.

**Methods:**

We included 5,614 pregnancies from three hospitals in China. For each pregnancy, the earliest eligible uric acid measurement before 24 weeks of gestation was retained. Uric acid was analysed in quartiles and per 10 μmol/L increase. Modified Poisson regression with robust variance estimated relative risks (RRs), restricted cubic splines assessed dose-response patterns, and sensitivity analyses examined the stability of the findings.

**Results:**

GDM occurred in 1,187 pregnancies (21.1%) and HDP in 275 pregnancies (4.9%). Compared with Q1, Q4 was associated with higher risk of GDM (adjusted RR, 1.54; 95% CI, 1.34 to 1.77) and HDP (adjusted RR, 1.89; 95% CI, 1.33 to 2.67). Each 10 μmol/L increase in uric acid was associated with GDM (adjusted RR, 1.03; 95% CI, 1.02 to 1.04) and HDP (adjusted RR, 1.04; 95% CI, 1.02 to 1.06). Spline analyses showed modest evidence of nonlinearity for GDM but not for HDP.

**Discussion:**

Maternal serum uric acid measured before 24 weeks of gestation was associated with subsequent GDM, with a related association observed for HDP. Uric acid may complement established maternal risk factors but should not be interpreted as a standalone screening test.

## Introduction

Gestational diabetes mellitus (GDM) is a common metabolic complication of pregnancy and is associated with obstetric complications, neonatal morbidity, and later cardiometabolic disease in both mothers and offspring (1–3). In routine care, diagnostic testing is usually performed at 24 to 28 gestational weeks (4). This timing creates a clinical gap: metabolic risk may already be present before formal diagnosis, but most women have not yet undergone diagnostic testing. A biochemical marker measured before this window is useful only if it adds interpretable risk information alongside established maternal factors and does not substitute for oral glucose tolerance testing.

Serum uric acid is frequently measured in antenatal clinical chemistry testing. Although traditionally viewed as a product of purine metabolism, higher uric acid concentrations have been linked to insulin resistance, oxidative stress, inflammation, endothelial dysfunction, renal metabolic stress, and cardiometabolic disease (5, 6). These processes are directly relevant to impaired glucose regulation during pregnancy and to abnormal maternal metabolic adaptation. Uric acid has also been reported in studies of female reproductive disorders and pregnancy complications, supporting its role as a marker of maternal metabolic and vascular status rather than as a GDM specific biomarker (7).

Evidence linking maternal uric acid to GDM has accumulated. Two meta-analyses reported a positive association between higher maternal serum uric acid and GDM risk (8, 9), and a large Chinese cohort published in 2022 found that early pregnancy serum uric acid was associated with subsequent GDM (10). The unresolved issue is how this association should be interpreted in clinical research. Important considerations include the gestational timing of uric acid measurement, the shape of the dose-response relationship, adjustment for adiposity and preexisting disease, and consistency across hospitals with different testing practices and outcome frequencies.

Hypertensive disorders of pregnancy (HDP) offer a related cardiometabolic comparison. GDM and hypertensive pregnancy disorders are clinically distinct, but both are linked to insulin resistance, adiposity, endothelial dysfunction, inflammation, and abnormal vascular adaptation (1, 3, 11, 12). Examining HDP alongside GDM can therefore clarify whether early maternal serum uric acid is more consistent with glucose related risk alone or with broader maternal metabolic and vascular susceptibility. In this multicentre cohort from three hospitals in China, we examined the association between maternal serum uric acid measured before 24 weeks of gestation and GDM during the index pregnancy. HDP was evaluated as a related cardiometabolic outcome. Uric acid was analysed by quartiles and per 10 μmol/L increase, with dose-response, sensitivity, hospital-stratified, and supplementary analyses used to evaluate robustness to analytical assumptions and consistency across hospital settings.

## Materials and methods

### Study design and data sources

This multicentre retrospective cohort study used routinely collected prenatal and obstetric clinical data from three participating hospitals in China. The participating hospitals were The People’s Hospital of Lincang, Yunnan, Shanghai Tenth People’s Hospital, and Hubei Maternal and Child Health Hospital. The study examined whether maternal serum uric acid measured before 24 weeks of gestation was associated with GDM during the index pregnancy. HDP was evaluated as a related cardiometabolic pregnancy outcome.

At all three centres, serum uric acid was routinely included in antenatal clinical chemistry testing, generally together with serum creatinine, rather than measured under a study-mandated protocol or ordered specifically because of suspected GDM or hyperuricaemia. The study was reported with reference to the STROBE guidance for observational studies (13).

### Study population

The source dataset included 12,177 processed uric acid-related clinical records. Records were screened for valid gestational age at uric acid measurement and for eligibility of uric acid measurement before 24 weeks of gestation. Records with missing gestational age, implausible gestational age, or no eligible uric acid measurement before 24 weeks of gestation were excluded at the record level. For pregnancies with more than one eligible uric acid measurement before 24 weeks, the earliest eligible measurement was retained to construct a pregnancy-level analytic dataset.

After eligible records were collapsed to unique pregnancies, 5,625 pregnancies remained. Eleven pregnancies were excluded because of missing covariates required for the primary model, resulting in a primary analytic cohort of 5,614 pregnancies. Variable availability and cohort construction are shown in **Supplementary eTables 2** and **3**.

The primary analytic cohort included pregnancies with available maternal serum uric acid before 24 weeks of gestation, valid gestational age at measurement, ascertainable GDM and HDP outcomes, and complete data for covariates included in the primary adjusted model. No multiple imputation was performed because missingness in model-required variables was low.

To describe potential selection related to the timing of uric acid testing, baseline characteristics were compared between pregnancies with any uric acid measurement before 24 weeks of gestation and pregnancies with uric acid measurements only at or after 24 weeks of gestation. Standardized mean differences were used for this descriptive comparison, which was reconstructed from the raw uric acid-related record table rather than from the primary analytic cohort. Exposure.

The exposure was maternal serum uric acid measured before 24 weeks of gestation. Serum uric acid was measured in local clinical laboratories using uricase-based enzymatic colorimetric assays and was reported in μmol/L. Centre-specific analyser platforms, reagent systems, reference intervals, calibration procedures, and internal quality-control practices are summarised in **Supplementary eTable 1**. Uric acid was analysed in two ways. First, it was categorized into quartiles based on its distribution in the primary analytic cohort: Q1, ≤ 185 μmol/L; Q2, > 185 to ≤ 216 μmol/L; Q3, > 216 to ≤ 251 μmol/L; and Q4, > 251 μmol/L. Second, uric acid was analysed as a continuous variable per 10 μmol/L increase. The lowest quartile was used as the reference group for categorical analyses.

### Outcomes

The primary outcome was GDM diagnosed during the index pregnancy. GDM was harmonised according to 75 g oral glucose tolerance test criteria, with one or more abnormal values considered diagnostic: fasting plasma glucose ≥ 5.1 mmol/L, one-hour plasma glucose ≥ 10.0 mmol/L, or 2-hour plasma glucose ≥ 8.5 mmol/L (4).

The secondary outcome was HDP. HDP was defined as gestational hypertension, preeclampsia, or eclampsia recorded during the index pregnancy, consistent with contemporary clinical classification of hypertensive disorders in pregnancy (14). GDM and HDP ascertainment was based on prenatal screening records, diagnostic records, and obstetric inpatient diagnosis records available at the participating centres.

### Covariates

The primary adjusted model included hospital, gestational age at uric acid measurement, maternal age, prepregnancy body mass index, urban residence, gravidity, parity, pregestational diabetes, chronic hypertension, thyroid disease, polycystic ovary syndrome, and kidney disease. These variables were selected because of their clinical relevance to maternal metabolic status, pregnancy complications, or potential differences in measurement and outcome ascertainment across centres. The same covariate set was used for GDM and HDP models unless otherwise specified.

### Statistical analysis

Baseline characteristics were summarized according to quartiles of maternal uric acid. Continuous variables were reported as mean and standard deviation or median and interquartile range, depending on distribution. Categorical variables were reported as number and percentage.

Associations between maternal uric acid and GDM or HDP were estimated using modified Poisson regression models with robust variance to obtain relative risks and 95% confidence intervals (15). Uric acid was modelled both as quartiles and as a continuous exposure per 10 μmol/L increase. Tests for trend across quartiles were performed by entering the quartile variable as an ordinal term in the model. Crude and adjusted estimates were reported for the primary association analyses.

Restricted cubic spline models were fitted within the modified Poisson regression framework with robust variance to evaluate dose-response patterns between maternal uric acid and each outcome (16). The spline models were adjusted for the same covariates as the primary analyses. Four knots were placed at the 5th, 35th, 65th, and 95th percentiles of maternal uric acid, with the median value used as the reference. Overall associations and departures from linearity were assessed using robust Wald tests.

Several sensitivity analyses were conducted to evaluate the stability of the association between maternal uric acid and GDM. First, analyses were restricted to pregnancies with uric acid measured before 20 weeks of gestation. Second, analyses were repeated after excluding the lowest and highest 1% of uric acid values. Third, pregnancies identified as multiple gestations were excluded. Fourth, the primary models were repeated after excluding pregnancies with pregestational diabetes to address potential ambiguity in the interpretation of incident GDM. Pregestational diabetes was included as an adjustment covariate in the primary model but was omitted from the restricted model because no such pregnancies remained. Uric acid quartile cut points were kept the same as in the primary analysis to improve comparability across models.

To examine consistency across centres, hospital-specific distributions of uric acid and outcome frequencies were summarized. Associations between maternal uric acid and outcomes were then estimated separately within each hospital. Interaction by hospital was assessed by adding a multiplicative interaction term between maternal uric acid and hospital to the pooled model. Leave-one-hospital-out analyses were also performed by repeating the primary adjusted models after excluding each hospital in turn.

Additional supplementary analyses included adjustment for ethnicity in the subcohort with available ethnicity data, exploratory interaction analyses between maternal uric acid and ethnicity, comparison of models with and without prepregnancy BMI adjustment, and interaction testing between maternal uric acid and gestational age at uric acid measurement.

Additional exploratory subgroup analyses were performed according to prepregnancy BMI (< 24 vs ≥ 24 kg/m²), maternal age (< 35 vs ≥ 35 years), PCOS status, and gravidity (1 vs ≥ 2). Maternal serum uric acid was modelled continuously per 10 μmol/L increase. Subgroup-specific associations were estimated using modified Poisson regression with robust variance, and effect modification was assessed using multiplicative interaction terms. The variable used to define each subgroup was omitted from the corresponding subgroup-specific adjustment model.

Exploratory prediction analyses compared established maternal risk factor models with and without maternal uric acid using apparent AUC, bootstrap optimism-corrected AUC, repeated 10-fold cross-validated AUC, and Brier score (17, 18). Category-free continuous net reclassification improvement and integrated discrimination improvement were additionally calculated to quantify the incremental predictive value of maternal serum uric acid. The 95% confidence intervals were estimated using 1,000 bootstrap resamples. Category-based NRI was not calculated because validated clinical risk categories for early-pregnancy prediction of GDM or HDP were unavailable. These prediction analyses were considered exploratory and were not used to define uric acid as a standalone screening test.

All statistical analyses were performed using R version 4.3.2. A two-sided P value < 0.05 was considered statistically significant. For secondary and exploratory analyses, P values were interpreted together with effect estimates, confidence intervals, and consistency across sensitivity analyses.

## Results

### Cohort construction

**Figure 1:**
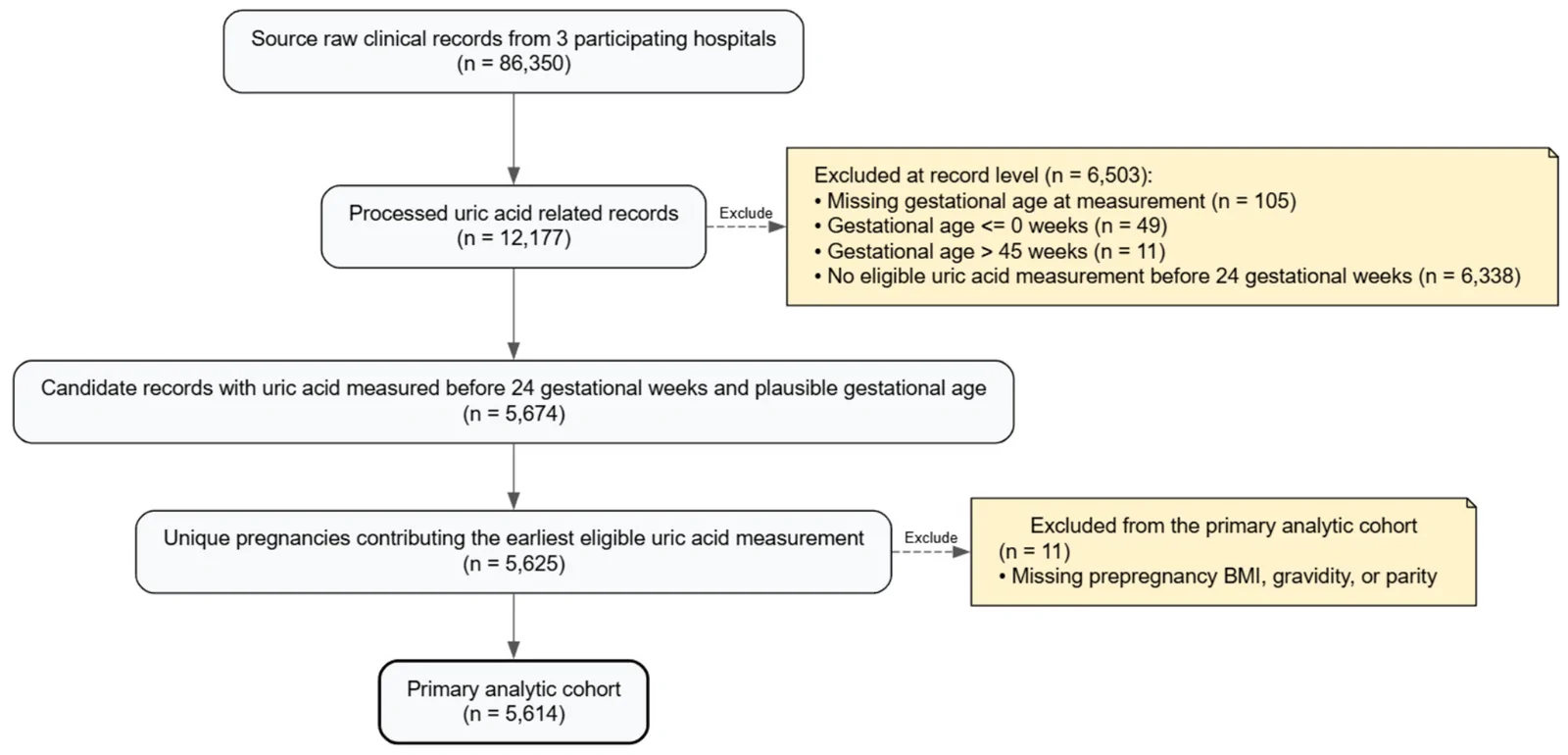
Flow diagram of cohort construction. The source dataset contained 12,177 uric acid-related clinical records. After excluding records without a plausible gestational age or without an eligible maternal serum uric acid measurement before 24 weeks of gestation, 5,674 records remained. For pregnancies with more than one eligible measurement, the earliest measurement was retained. After collapsing records to unique pregnancies, 5,625 pregnancies remained. Eleven pregnancies with missing covariates required for the primary adjusted model were excluded, resulting in a final analytic cohort of 5,614 pregnancies. UA, uric acid.

Among 12,177 processed uric acid-related clinical records, 5,674 records had a plausible gestational age and at least one uric acid measurement before 24 weeks of gestation. After retaining the earliest eligible uric acid measurement for each pregnancy, 5,625 unique pregnancies remained. Eleven pregnancies were excluded because of missing covariates required for the primary model, resulting in a primary analytic cohort of 5,614 pregnancies (**Figure 1**; **Supplementary eTables 2**, **3**). A descriptive comparison of pregnancies with at least one uric acid measurement before 24 weeks and those with uric acid measurements only at or after 24 weeks is provided in **Supplementary eTable 4**.

### Baseline characteristics according to maternal uric acid quartiles

The median gestational age at uric acid measurement was 14.4 weeks. Across increasing uric acid quartiles, prepregnancy BMI increased from 21.3 kg/m² in Q1 to 22.5 kg/m² in Q4. Pregestational diabetes, chronic hypertension, polycystic ovary syndrome, and kidney disease were more frequent in Q4 than in Q1. GDM occurred in 1,187 pregnancies, increasing from 18.3% in Q1 to 27.6% in Q4. HDP occurred in 275 pregnancies, increasing from 3.3% in Q1 to 7.4% in Q4 (**Table 1**).

**Table 1:** Baseline characteristics and pregnancy outcomes according to quartiles of maternal serum uric acid measured before 24 weeks of gestation.

Characteristic	Overall(N=5614)	Q1 (N=1434)	Q2 (N=1413)	Q3 (N=1368)	Q4 (N=1399)
		UA≤185	185< UA≤216	216< UA≤251	251< UA
Hospital for giving birth, n. (%)					
The People's Hospital of Lincang, Yunnan	1741 (31.0)	659 (46.0)	395 (28.0)	316 (23.1)	371 (26.5)
Shanghai Tenth People's Hospital	1325 (23.6)	272 (19.0)	360 (25.5)	388 (28.4)	305 (21.8)
Wuhan Maternal and Child Health	2548 (45.4)	503 (35.1)	658 (46.6)	664 (48.5)	723 (51.7)
Gestational age at UA measurement, weeks	14.4 [12.0, 16.7]	13.4 [10.7, 16.0]	14.6 [12.1, 16.7]	15.1 [12.4, 17.0]	14.9 [12.1, 17.0]
Maternal age, years	30.39 (3.96)	30.4 (4.05)	30.3 (3.90)	30.3 (3.87)	30.5 (4.00)
Prepregnancy BMI, kg/m^2^	21.88 (3.34)	21.3 (3.05)	21.7 (3.10)	22.1 (3.42)	22.5 (3.65)
Urban residence, n. (%)	4882 (87.0)	1249 (87.1)	1214 (85.9)	1188 (86.8)	1231 (88.0)
Gravidity, n. (%)					
1	3533 (62.9)	817 (57.0)	887 (62.8)	903 (66.0)	926 (66.2)
≥2	2081 (37.1)	617 (43.0)	526 (37.2)	465 (34.0)	473 (33.8)
Parity, n. (%)					
0	4226 (75.3)	1000 (69.7)	1072 (75.9)	1062 (77.6)	1092 (78.1)
1	1199 (21.4)	387 (27.0)	298 (21.1)	258 (18.9)	256 (18.3)
≥2	189 (3.4)	47 (3.3)	43 (3.0)	48 (3.5)	51 (3.6)
Pre-existing diseases, n. (%)					
Pregestational diabetes	40 (0.7)	5 (0.3)	6 (0.4)	8 (0.6)	21 (1.5)
Chronic hypertension	46 (0.8)	4 (0.3)	10 (0.7)	10 (0.7)	22 (1.6)
Thyroid disease	862 (15.4)	224 (15.6)	220 (15.6)	196 (14.3)	222 (15.9)
Polycystic ovary syndrome	80 (1.4)	11 (0.8)	16 (1.1)	18 (1.3)	35 (2.5)
Kidney disease	51 (0.9)	15 (1.0)	5 (0.4)	8 (0.6)	23 (1.6)
Pregnancy outcomes, n. (%)					
Gestational diabetes mellitus	1187 (21.1)	263 (18.3)	249 (17.6)	289 (21.1)	386 (27.6)
Hypertensive disorders of pregnancy	275 (4.9)	47 (3.3)	57 (4.0)	67 (4.9)	104 (7.4)

Data are presented as mean (SD), median [IQR], or n. (%). UA indicates uric acid.

### Association between maternal uric acid and GDM

In the adjusted model, higher maternal uric acid before 24 weeks of gestation was associated with higher GDM risk. Compared with Q1, the adjusted RR was 1.05 (95% CI, 0.90 to 1.22) for Q2, 1.27 (95% CI, 1.10 to 1.48) for Q3, and 1.54 (95% CI, 1.34 to 1.77) for Q4. The trend across quartiles was statistically significant. When uric acid was modelled continuously, each 10 μmol/L increase was associated with higher GDM risk (adjusted RR, 1.03; 95% CI, 1.02 to 1.04) (**Table 2**).

**Table 2:** Associations of maternal uric acid measured before 24 weeks of gestation with gestational diabetes mellitus and hypertensive disorders of pregnancy.

Outcome	Exposure	Crude RR (95% CI)	Adjusted RR (95% CI)	Crude P value	Adjusted P value
GDM	Q1	Reference	Reference		
	Q2 vs Q1	0.96 (0.82, 1.12)	1.05 (0.90, 1.22)	0.618	0.560
	Q3 vs Q1	1.15 (0.99, 1.34)	1.27 (1.10, 1.48)	0.064	0.002
	Q4 vs Q1	1.50 (1.31, 1.73)	1.54 (1.34, 1.77)	<0.001	<0.001
	*P* for trend			<0.001	<0.001
	Per 10 μmol/L increase	1.03 (1.02, 1.04)	1.03 (1.02, 1.04)	<0.001	<0.001
HDP	Q1	Reference	Reference		
	Q2 vs Q1	1.23 (0.84, 1.80)	1.12 (0.76, 1.64)	0.283	0.573
	Q3 vs Q1	1.49 (1.04, 2.15)	1.31 (0.90, 1.90)	0.031	0.156
	Q4 vs Q1	2.27 (1.62, 3.18)	1.89 (1.33, 2.67)	<0.001	<0.001
	*P* for trend			<0.001	<0.001
	Per 10 μmol/L increase	1.05 (1.04, 1.07)	1.04 (1.02, 1.06)	<0.001	<0.001

Adjusted models included hospital, gestational age at uric acid measurement, maternal age, prepregnancy body mass index, urban residence, gravidity, parity, pregestational diabetes, chronic hypertension, thyroid disease, polycystic ovary syndrome, and kidney disease. GDM indicates gestational diabetes mellitus; HDP, hypertensive disorders of pregnancy; RR, relative risk.

### Association between maternal uric acid and HDP

A similar direction of association was observed for HDP. Compared with Q1, the adjusted RR was 1.12 (95% CI, 0.76 to 1.64) for Q2, 1.31 (95% CI, 0.90 to 1.90) for Q3, and 1.89 (95% CI, 1.33 to 2.67) for Q4. Each 10 μmol/L increase in uric acid was associated with higher HDP risk (adjusted RR, 1.04; 95% CI, 1.02 to 1.06) (**Table 2**).

### Dose response analyses

**Figure 2:**
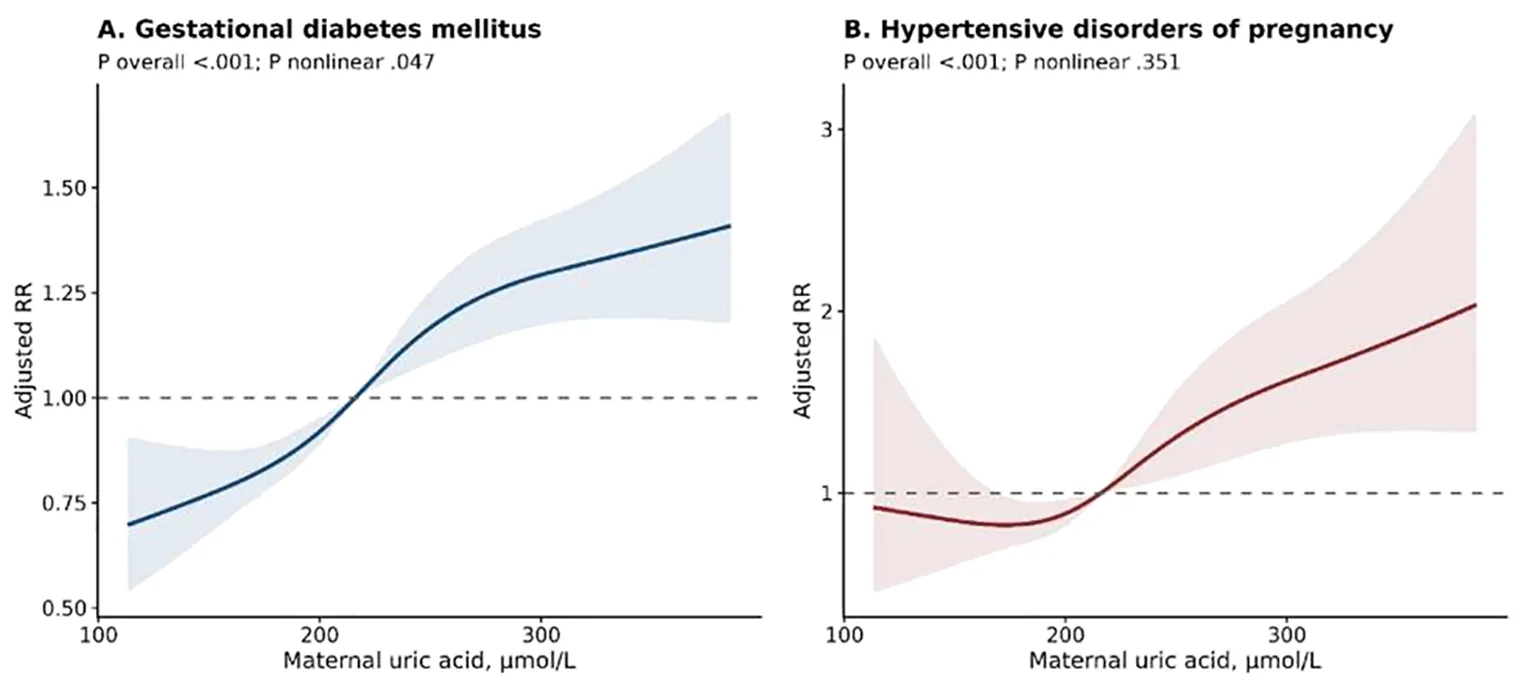
Restricted cubic spline associations of maternal serum uric acid measured before 24 weeks of gestation with gestational diabetes mellitus and hypertensive disorders of pregnancy. Restricted cubic spline analyses included 5,614 pregnancies and were fitted within modified Poisson regression models with robust variance estimation. The models were adjusted for hospital, gestational age at uric acid measurement, maternal age, prepregnancy body mass index, urban residence, gravidity, parity, pregestational diabetes, chronic hypertension, thyroid disease, polycystic ovary syndrome, and kidney disease. Four knots were placed at the 5th, 35th, 65th, and 95th percentiles of maternal serum uric acid, and the median uric acid concentration was used as the reference. Panel **(A)** shows the association with gestational diabetes mellitus, and panel **(B)** shows the association with hypertensive disorders of pregnancy. Solid lines indicate adjusted relative risks, shaded areas indicate 95% confidence intervals, and the horizontal reference line indicates a relative risk of 1.0. Overall associations and departures from linearity were assessed using robust Wald tests. For gestational diabetes mellitus, *P* for overall association was <0.001 and *P* for nonlinearity was 0.047. For hypertensive disorders of pregnancy, *P* for overall association was <0.001 and P for nonlinearity was 0.351. All tests were two sided, and P < 0.05 was considered statistically significant. CI, confidence interval; GDM, gestational diabetes mellitus; HDP, hypertensive disorders of pregnancy; RR, relative risk; UA, uric acid.

Restricted cubic spline analyses showed significant overall associations between maternal uric acid and both outcomes. There was modest evidence of nonlinearity for GDM (*P* for overall association <0.001; *P* for nonlinearity = 0.047), whereas no evidence of nonlinearity was observed for HDP (*P* for overall association <0.001; *P* for nonlinearity = 0.351) (**Figure 2**).

### Sensitivity analyses for GDM

GDM results were similar across analyses restricted to uric acid measurements before 20 weeks of gestation, excluding the lowest and highest 1% of uric acid values, and excluding multiple pregnancies. In the analysis excluding pregnancies with pregestational diabetes, 5,574 pregnancies remained. The adjusted RR for Q4 versus Q1 was 1.55 (95% CI, 1.35 to 1.78), and the adjusted RR per 10 μmol/L increase was 1.03 (95% CI, 1.02 to 1.04) (**Table 3**; **Supplementary eTables 5**–**7**).

**Table 3:** Sensitivity analyses of the association between maternal serum uric acid and gestational diabetes mellitus.

Analysis	Q4 vs Q1 adjusted RR (95% CI)	*P* value	Per 10 μmol/L increase, adjusted RR (95% CI)	*P* value
Primary cohort	1.54 (1.34 to 1.77)	<0.001	1.03 (1.02 to 1.04)	<0.001
Restricted to uric acid measurements before 20 weeks of gestation	1.53 (1.32 to 1.76)	<0.001	1.03 (1.02 to 1.04)	<0.001
Excluding the lowest and highest 1% of uric acid values	1.50 (1.30 to 1.73)	<0.001	1.03 (1.02 to 1.04)	<0.001
Excluding multiple gestations	1.53 (1.33 to 1.76)	<0.001	1.03 (1.02 to 1.04)	<0.001
Excluding pregnancies with pregestational diabetes	1.55 (1.35 to 1.78)	<0.001	1.03 (1.02 to 1.04)	<0.001

Relative risks and 95% confidence intervals were estimated using modified Poisson regression models with robust variance. Quartile cut points were fixed according to the distribution in the primary analytic cohort. Models were adjusted for hospital, gestational age at uric acid measurement, maternal age, prepregnancy body mass index, urban residence, gravidity, parity, pregestational diabetes, chronic hypertension, thyroid disease, polycystic ovary syndrome, and kidney disease. Pregestational diabetes was omitted from the model excluding pregnancies with pregestational diabetes because no such pregnancies remained. Only the Q4 versus Q1 and continuous estimates are presented in the main table; complete quartile-specific results are provided in **Supplementary eTables 5–7**. CI, confidence interval; GDM, gestational diabetes mellitus; RR, relative risk.

### Hospital stratified and supplementary analyses

In hospital-stratified analyses, the adjusted RR for GDM per 10 μmol/L increase in maternal uric acid was above 1.00 in all three hospitals. The adjusted RRs were 1.03 in Lincang, 1.02 in Hubei Maternal and Child Health Hospital, and 1.05 in Shanghai Tenth People’s Hospital. The interaction by hospital was not statistically significant (*P* for interaction = 0.288) (**Supplementary eTable 9**). Leave-one-hospital-out analyses gave estimates similar to the primary analysis (**Supplementary eTable 10**). Additional analyses adjusting for ethnicity, comparing models with and without prepregnancy BMI adjustment, and testing interaction with gestational age at uric acid measurement are shown in **Supplementary eTables 11**–**15**. Exploratory prediction analyses showed small increases in discrimination after adding maternal uric acid to established maternal risk factors (**Supplementary eTables 16a**, **16b**). The continuous NRI was 0.174 (95% CI, 0.112 to 0.240) for GDM and 0.236 (95% CI, 0.107 to 0.364) for HDP. The corresponding IDI values were 0.010 (95% CI, 0.005 to 0.016) and 0.004 (95% CI, 0.001 to 0.009), respectively (**Supplementary eTable 16c**). The small absolute IDI values indicated limited incremental improvement in predictive discrimination. In exploratory subgroup analyses, the associations were generally consistent across subgroups defined by prepregnancy BMI, maternal age, PCOS status, and gravidity, with no evidence of effect modification for either outcome (all *P* values for interaction > 0.05; **Supplementary eTable 17**).

## Discussion

In this multicentre retrospective cohort study of 5,614 pregnancies, higher maternal serum uric acid measured before 24 weeks of gestation was associated with higher risk of subsequent GDM, with a related association observed for HDP. The association with GDM was graded across uric acid quartiles, with an adjusted RR of 1.54 for the highest versus the lowest quartile, whereas the association per 10 μmol/L increase was modest. Restricted cubic spline analyses showed broadly increasing associations for both outcomes, with modest evidence of nonlinearity for GDM but not for HDP. The findings remained generally stable across sensitivity analyses.

Previous studies have reported that higher maternal serum uric acid is associated with a greater risk of GDM, including two meta-analyses published in 2023 and a large Chinese cohort study published in 2022 (8–10). Our findings are consistent with these reports. By restricting uric acid measurements to before 24 weeks of gestation, the present study focused on a period preceding routine GDM diagnosis. We also accounted for gestational age at measurement and prepregnancy BMI, examined dose-response patterns, and assessed consistency across three hospitals. Because GDM is conventionally diagnosed using an oral glucose tolerance test at 24–28 gestational weeks (4), an earlier uric acid measurement is better viewed as a risk marker than as a diagnostic test or an alternative to standard GDM screening.

Several biological pathways may help explain the observed association. Pregnancy is normally accompanied by a progressive decline in insulin sensitivity, which must be matched by greater β-cell insulin secretion. Higher uric acid may make this metabolic adaptation more difficult by promoting oxidative stress and low-grade inflammation, both of which can impair insulin signaling, worsen peripheral insulin resistance, and increase the compensatory demand on β cells. Reduced nitric oxide bioavailability and endothelial dysfunction may further impair the metabolic and vascular adaptations of pregnancy (5, 6). Nutrient sensing mediated by mTOR may provide a plausible link between metabolic stress and pancreatic β cell adaptation. Experimental studies have linked the Rab1A, mTORC1, and PDX1 pathway, as well as PDX1 phosphorylation mediated by mTORC1, to nutrient sensing, insulin production, and pancreatic β cell function (19, 20). mTOR signaling also interacts with AMPK and with signaling through insulin and insulin-like growth factor 1 during metabolic adaptation (21). However, mTOR activity was not measured in the present study, and whether maternal uric acid influences this pathway in human GDM remains uncertain. A conceptual summary of the proposed metabolic and vascular pathways is provided in **Supplementary eFigure 1**.

The associations were generally consistent across subgroups defined by prepregnancy BMI, maternal age, PCOS status, and gravidity, with no evidence of effect modification. For GDM, adding uric acid to established maternal risk factors yielded positive continuous NRI and IDI estimates, but the gains in AUC and the absolute IDI values were small. Clinically, this indicates that a marker may be associated with subsequent GDM without providing sufficient accuracy for use as a standalone screening test. In a recent prospective study, Tigli et al. evaluated nine routinely available metabolic and inflammatory indices at 10 to 14 weeks of gestation, but all had AUCs below 0.70 for early GDM prediction (22). Our findings point in the same direction. Uric acid measured in early pregnancy may modestly refine a broader maternal risk assessment, but it should be regarded as an adjunct rather than a standalone screening marker and cannot replace the standard oral glucose tolerance test at 24 to 28 gestational weeks.

The parallel association with HDP places the GDM finding in a broader cardiometabolic context. Higher uric acid may reflect a shared background of metabolic and vascular vulnerability relevant to both conditions, including insulin resistance, inflammation, and endothelial dysfunction (1, 3, 11, 12). However, the two outcomes should not be assumed to arise through the same biological pathway. In this study, HDP was evaluated as a related outcome to help contextualise the GDM association rather than as a second primary endpoint.

Several checks gave similar results. Restricting uric acid measurements to before before 20 weeks of gestation, excluding extreme values, and excluding multiple pregnancies did not materially change the associations. Because pregestational diabetes complicates the interpretation of incident GDM, we also repeated the analysis after excluding these pregnancies, and the estimates remained essentially unchanged. Hospital specific RR estimates for GDM were greater than 1.00 in all three hospitals, with no evidence of interaction by hospital. These findings reduce, although do not eliminate, concern that the results were driven by measurement timing, extreme values, multiple gestation, pregestational diabetes, or a single hospital.

A few limitations deserve emphasis. First, the retrospective design precludes causal inference. Although serum uric acid was routinely included in antenatal clinical chemistry testing at all three centres, not every pregnancy had an available measurement before 24 weeks of gestation. Selection related to the timing and availability of early measurements therefore cannot be excluded. Second, analyser platforms, reagent systems, and reference intervals differed across centres. All laboratories used uricase-based assays and followed local calibration and quality control procedures, but residual interlaboratory variation remains possible despite adjustment for centre and centre-specific analyses. Third, residual confounding remains possible because diet, physical activity, medication use, family history of diabetes, and several metabolic markers were unavailable. Diagnosed kidney disease was included in the models, but comparable serum creatinine values were not consistently available. Mild or unrecorded renal dysfunction may therefore have contributed to residual confounding. Finally, a single uric acid measurement may not capture changes across pregnancy, and findings from three Chinese hospitals may not generalise to other populations. In conclusion, maternal serum uric acid measured before 24 weeks of gestation was associated with subsequent GDM, with a related association observed for HDP. Although uric acid may modestly complement risk assessment based on established maternal factors, its incremental predictive value was limited, and it should not replace standard GDM screening with an oral glucose tolerance test at 24 to 28 gestational weeks.

## Data Availability

The raw data supporting the conclusions of this article will be made available by the authors, without undue reservation.
