# Correction: Maternal serum uric acid before 24 weeks of gestation and subsequent gestational diabetes mellitus: a multicentre retrospective cohort study

**DOI:** 10.3389/fendo.2026.1950401

**Published:** 2026-08-20

**Authors:** Qinquan Cheng, Yuan Chen, Wenkai Zhang, Saijie Ma, Hua Tian, Shijie Wu, Yuan Jiang, Xiangdong Huang, Hanping Yang, Mingming Ding, Minghao Kong

**Affiliations:** 1 Department of Clinical Laboratory Medicine, Shanghai Tenth People’s Hospital, Tongji University School of Medicine, Shanghai, China; 2 Department of Obstetrics and Gynecology, The People’s Hospital of Lincang, Lincang, Yunnan, China; 3 Department of Medical Intensive Care Unit, Maternal and Child Health Hospital of Hubei Province, Tongji Medical College, Huazhong University of Science and Technology, Wuhan, Hubei, China; 4 Center for Nephrology & Metabolomics, Division of Nephrology, Shanghai Tenth People’s Hospital, Tongji University School of Medicine, Shanghai, China; 5 Department of Obstetrics, Gong’an County Hospital, Jingzhou, Hubei, China; 6 Department of Nursing, Shanghai Tenth People’s Hospital, Tongji University School of Medicine, Shanghai, China; 7 Department of Medical Intensive Care Unit, The People’s Hospital of Lincang, Lincang, Yunnan, China; 8 Department of Information Management, Shanghai Tenth People’s Hospital, Tongji University School of Medicine, Shanghai, China; 9 Department of Emergency Medicine, Shanghai Tenth People’s Hospital, Tongji University School of Medicine, Shanghai, China

**Keywords:** cardiometabolic risk, endocrine metabolism, gestational diabetes mellitus, hypertensive disorders of pregnancy, maternal uric acid, pregnancy, retrospective cohort

Affiliation “Center for Nephrology & Metabolomics, Division of Nephrology, Shanghai Tenth People’s Hospital, Tongji University School of Medicine, Shanghai, China” was omitted from the paper. This affiliation has now been added for authors Saijie Ma and Shijie Wu.

Affiliation “Kidney and Metabolism Center, Shanghai Tenth People’s Hospital, Tongji University School of Medicine, Shanghai, China” was erroneously included in the paper. This affiliation has now been removed for authors Saijie Ma and Shijie Wu.

There was a mistake in **Table 1** as published. A subrow label under “Parity” was incorrectly labels as “≥2≥2”. It has been corrected to read “≥2”. 

The corrected **Table 1** appears below.

**Table 1:** Baseline characteristics and pregnancy outcomes according to quartiles of maternal serum uric acid measured before 24 weeks of gestation.

Characteristic	Overall(N=5614)	Q1 (N=1434)	Q2 (N=1413)	Q3 (N=1368)	Q4 (N=1399)
		UA≤185	185< UA≤216	216< UA≤251	251< UA
Hospital for giving birth, n. (%)					
The People's Hospital of Lincang, Yunnan	1741 (31.0)	659 (46.0)	395 (28.0)	316 (23.1)	371 (26.5)
Shanghai Tenth People's Hospital	1325 (23.6)	272 (19.0)	360 (25.5)	388 (28.4)	305 (21.8)
Wuhan Maternal and Child Health	2548 (45.4)	503 (35.1)	658 (46.6)	664 (48.5)	723 (51.7)
Gestational age at UA measurement, weeks	14.4 [12.0, 16.7]	13.4 [10.7, 16.0]	14.6 [12.1, 16.7]	15.1 [12.4, 17.0]	14.9 [12.1, 17.0]
Maternal age, years	30.39 (3.96)	30.4 (4.05)	30.3 (3.90)	30.3 (3.87)	30.5 (4.00)
Prepregnancy BMI, kg/m^2^	21.88 (3.34)	21.3 (3.05)	21.7 (3.10)	22.1 (3.42)	22.5 (3.65)
Urban residence, n. (%)	4882 (87.0)	1249 (87.1)	1214 (85.9)	1188 (86.8)	1231 (88.0)
Gravidity, n. (%)					
1	3533 (62.9)	817 (57.0)	887 (62.8)	903 (66.0)	926 (66.2)
≥2	2081 (37.1)	617 (43.0)	526 (37.2)	465 (34.0)	473 (33.8)
Parity, n. (%)					
0	4226 (75.3)	1000 (69.7)	1072 (75.9)	1062 (77.6)	1092 (78.1)
1	1199 (21.4)	387 (27.0)	298 (21.1)	258 (18.9)	256 (18.3)
≥2	189 (3.4)	47 (3.3)	43 (3.0)	48 (3.5)	51 (3.6)
Pre-existing diseases, n. (%)					
Pregestational diabetes	40 (0.7)	5 (0.3)	6 (0.4)	8 (0.6)	21 (1.5)
Chronic hypertension	46 (0.8)	4 (0.3)	10 (0.7)	10 (0.7)	22 (1.6)
Thyroid disease	862 (15.4)	224 (15.6)	220 (15.6)	196 (14.3)	222 (15.9)
Polycystic ovary syndrome	80 (1.4)	11 (0.8)	16 (1.1)	18 (1.3)	35 (2.5)
Kidney disease	51 (0.9)	15 (1.0)	5 (0.4)	8 (0.6)	23 (1.6)
Pregnancy outcomes, n. (%)					
Gestational diabetes mellitus	1187 (21.1)	263 (18.3)	249 (17.6)	289 (21.1)	386 (27.6)
Hypertensive disorders of pregnancy	275 (4.9)	47 (3.3)	57 (4.0)	67 (4.9)	104 (7.4)

Data are presented as mean (SD), median [IQR], or n. (%). UA indicates uric acid.

