# Supplementary material for: Maternal serum uric acid before 24 weeks of gestation and subsequent gestational diabetes mellitus: a multicentre retrospective cohort study
Source: Front Endocrinol (Lausanne). 2026 Jul 24;17:1896745. doi: 10.3389/fendo.2026.1896745 (PMC13449704; doi:10.3389/fendo.2026.1896745)
Supplement: Supplementary file 1 [file SupplementaryFile1.docx]

eTable 1. Center Specific Laboratory Methods, Outcome Ascertainment, and Ethics Approvals

**Center**

**UA Laboratory Method**

**GDM**

**HDP**

**Ethics Approval**

**Ascertainment**

Prenatal screening

**Ascertainment**

Prenatal or obstetric

The People’s

Hospital of

Beckman Coulter AU5800;

Autobio uric acid assay kit,

uricase method

Ethics Committee of The People’s Hospital

of Lincang; approval No. 伦理审 2025-080

（自-横）-01; file No. IEC/AE/61-02/2023-

01.0

and diagnosis records inpatient diagnosis

records

Lincang, Yunnan

Shanghai Tenth

Abbott Alinity c; uricase based

enzymatic assay

Prenatal screening

Prenatal or obstetric

Ethics Committee of Shanghai Tenth People’s

Hospital; approval No. SHSY-IEC-

6.0/25K213/P01; local ethics No. 25K213

Ethics Committee of Hubei Maternal and

Child Health Hospital; approval No. 2024

IEC (023)

People’s Hospital

and diagnosis records inpatient diagnosis

records

Wuhan Maternal

and Child Health

Hospital

Automated biochemical

analyzer; uricase based

enzymatic assay

Prenatal screening

Prenatal or obstetric

and diagnosis records inpatient diagnosis

records

**Notes:** Serum UA was measured in local clinical laboratories using uricase based assays and expressed in μmol/L. GDM was harmonized using

75 g OGTT criteria: fasting plasma glucose 5.1 mmol/L, 1 hour plasma glucose 10.0 mmol/L, or 2 hour plasma glucose 8.5 mmol/L, with 1 or

more abnormal values considered diagnostic. HDP was harmonized as gestational hypertension, preeclampsia, or eclampsia recorded during the

index pregnancy. Center specific ethics approval information is provided in the table.

eTable 2. Availability of Variables Used in the Primary Adjusted Model

**Variable**

**Role in analysis**

Exposure

**Available, No. (%)**

5625 (100.0)

5625 (100.0)

5625 (100.0)

5625 (100.0)

5625 (100.0)

5625 (100.0)

5619 (99.9)

**Missing, No. (%)**

0 (0.0)

Uric acid

Gestational age at uric acid measurement

GDM

Time window and covariate

Outcome

0 (0.0)

0 (0.0)

HDP

Outcome

0 (0.0)

Hospital

Covariate

0 (0.0)

Maternal age

Covariate

0 (0.0)

Prepregnancy BMI

Urban residence

Gravidity

Covariate

6 (0.1)

Covariate

5625 (100.0)

5621 (99.9)

0 (0.0)

Covariate

4 (0.1)

Parity

Covariate

5620 (99.9)

5 (0.1)

Pregestational diabetes

Chronic hypertension

Thyroid disease

Polycystic ovary syndrome

Kidney disease

Covariate

5625 (100.0)

5625 (100.0)

5625 (100.0)

5625 (100.0)

5625 (100.0)

0 (0.0)

Covariate

0 (0.0)

Covariate

0 (0.0)

Covariate

0 (0.0)

Covariate

0 (0.0)

**Note:** Missingness was summarized in the reconstructed harmonized analytic dataset before final complete-case restriction for the primary adjusted

model (n = 5625). The final reduction from 5625 pregnancies in the reconstructed harmonized analytic dataset to 5614 pregnancies in the primary

analytic cohort reflected exclusion of pregnancies with missing model-required covariates. Counts in this table are variable specific and are not

additive because missingness may overlap within the same pregnancy. No multiple imputation was performed.

eTable 3.Screening Summary for Construction of the Primary Analytic Cohort

**Step**

**N**

Processed uric acid related records

12177

105

49

Missing gestational age at measurement

Gestational age <= 0 weeks

Gestational age > 45 weeks

11

No eligible uric acid measurement before 24 gestational weeks

Candidate records with uric acid measured before 24 gestational weeks and plausible gestational age

Unique pregnancies contributing the earliest eligible uric acid measurement

Missing prepregnancy BMI

6338

5674

5625

6

Missing gravidity

4

Missing parity

5

Total pregnancies excluded before the primary analysis

Primary analytic cohort

11

5614

**Note**: This table summarizes both record-level screening and pregnancy-level construction of the primary analytic cohort. At the record level,

exclusions were applied sequentially to derive records with an eligible uric acid measurement before 24 gestational weeks and plausible gestational

age. Eligible records were then collapsed to unique pregnancies by retaining the earliest eligible uric acid measurement. Counts for missing

prepregnancy BMI, gravidity, and parity are condition specific and are not additive because missingness could overlap within the same pregnancy.

The final reduction from 5625 to 5614 reflects the union of pregnancies with missing model-required covariates.

eTable 4.Descriptive Comparison of Pregnancies With Early Versus Late-Only Uric Acid Measurements

**Characteristic**

**Overall, N = 5990**

**Early, n = 5625**

**Late-only, n = 365 SMD**

0.543

Hospital, n (%)

Lincang

1789 (29.9)

1398 (23.3)

2803 (46.8)

30.40 (3.97)

21.87 (3.34)

15.01 (4.76)

222.96 (55.55)

1744 (31.0)

1331 (23.7)

2550 (45.3)

30.39 (3.96)

21.89 (3.35)

14.23 (3.64)

221.18 (54.77)

45 (12.3)

Shanghai Tenth People’s Hospital

67 (18.4)

Wuhan Maternal and Child Health Hospital

253 (69.3)

Maternal age, years

30.58 (4.20)

21.62 (3.22)

27.02 (3.73)

250.44 (60.17)

0.046

0.082

3.472

0.509

0.011

Pre-pregnancy BMI, kg/m²

Gestational age at uric acid measurement, weeks

Uric acid, μmol/L

Urban residence, n (%)

No

783 (13.1)

734 (13.0)

49 (13.4)

Yes

5207 (86.9)

4891 (87.0)

316 (86.6)

Pre-existing diabetes in pregnancy, n (%)

0.013

0.033

0.052

0.066

No

5947 (99.3)

43 (0.7)

5585 (99.3)

40 (0.7)

362 (99.2)

3 (0.8)

Yes

Chronic hypertension, n (%)

No

5942 (99.2)

48 (0.8)

5579 (99.2)

46 (0.8)

363 (99.5)

2 (0.5)

Yes

Gestational diabetes mellitus, n (%)

No

4717 (78.7)

1273 (21.3)

4437 (78.9)

1188 (21.1)

280 (76.7)

85 (23.3)

Yes

Hypertensive disorders of pregnancy, n (%)

No

5702 (95.2)

288 (4.8)

5350 (95.1)

275 (4.9)

352 (96.4)

13 (3.6)

Yes

Continued eTable 4. Descriptive characteristics of pregnancies with any uric acid measurement before 24 gestational weeks versus pregnancies

with uric acid measurements only at or after 24 weeks

Gravidity, n (%)

0.196

1

3798 (63.4)

1141 (19.1)

1047 (17.5)

3536 (62.9)

1083 (19.3)

1002 (17.8)

262 (71.8)

58 (15.9)

45 (12.3)

2

≥3

Parity, n (%)

0.095

0

4515 (75.4)

1266 (21.2)

204 (3.4)

4230 (75.3)

1201 (21.4)

189 (3.4)

285 (78.1)

65 (17.8)

15 (4.1)

1

≥2

**Abbreviations:** BMI, body mass index; SMD, standardized mean difference. Pregnancies were classified as early if at least one uric acid measurement occurred

before 24 gestational weeks and as late-only if all available uric acid measurements occurred at or after 24 gestational weeks. This comparison was descriptive

and was reconstructed from the raw uric acid related records rather than from the primary analytic cohort. One clearly implausible parity value in the raw data

was treated as missing before tabulation.

eTable 5.Comparison of Primary and Sensitivity Analyses Restricted to Measurements Before 20 Gestational Weeks for the Associations of

Maternal Uric Acid With Gestational Diabetes Mellitus and Hypertensive Disorders of Pregnancy

**Part**

**Outcome Analysis**

GDM Primary analysis

**Exposure**

Q2 vs Q1

Q3 vs Q1

Q4 vs Q1

*P* for trend

Q2 vs Q1

**Adjusted RR (95% CI)**

1.05 (0.90, 1.22)

**P value**

0.560

Part A. UA quartiles

1.27 (1.10, 1.48)

0.002

1.54 (1.34, 1.77)

<0.001

<0.001

0.657

Restricted to measurements before 20

weeks

1.04 (0.89, 1.21)

Q3 vs Q1

Q4 vs Q1

*P* for trend

Q2 vs Q1

Q3 vs Q1

Q4 vs Q1

*P* for trend

Q2 vs Q1

1.26 (1.08, 1.46)

1.53 (1.32, 1.76)

0.003

<0.001

<0.001

0.573

HDP

Primary analysis

1.12 (0.76, 1.64)

1.31 (0.90, 1.90)

1.89 (1.33, 2.67)

0.156

<0.001

<0.001

0.694

Restricted to measurements before 20

weeks

1.08 (0.73, 1.62)

Q3 vs Q1

1.28 (0.87, 1.88)

1.91 (1.34, 2.74)

0.216

Q4 vs Q1

<0.001

<0.001

<0.001

*P* for trend

Per 10 μmol/L

increase

Part B. UA as a continuous

variable

GDM

HDP

Primary analysis

1.03 (1.02, 1.04)

1.03 (1.02, 1.04)

Restricted to measurements before 20

weeks

Per 10 μmol/L

increase

<0.001

<0.001

Primary analysis

Per 10 μmol/L increase

1.04 (1.02, 1.06)

Restricted to measurements before 20

weeks

Per 10 μmol/L increase

1.04 (1.02, 1.07)

<0.001

**Abbreviations:** GDM, gestational diabetes mellitus; HDP, hypertensive disorders of pregnancy; RR, relative risk. Adjusted models included

hospital, gestational age at uric acid measurement, maternal age, prepregnancy body mass index, urban residence, gravidity, parity, pregestational

diabetes, chronic hypertension, thyroid disease, polycystic ovary syndrome, and kidney disease.

eTable 6.Comparison of Primary and Sensitivity Analyses After Excluding the Lowest and Highest 1% of Uric Acid Values for the Associations

of Maternal Uric Acid With Gestational Diabetes Mellitus and Hypertensive Disorders of Pregnancy

**Part**

**Outcome**

**Analysis**

**Exposure**

Q2 vs Q1

Q3 vs Q1

Q4 vs Q1

*P* for trend

**Adjusted RR (95% CI) *P* value**

Part A. UA quartiles

GDM

Primary analysis

1.05 (0.90, 1.22)

1.27 (1.10, 1.48)

1.54 (1.34, 1.77)

0.560

0.002

<0.001

<0.001

0.753

Excluding the lowest and highest 1% Q2 vs Q1

1.03 (0.88, 1.20)

1.24 (1.07, 1.45)

1.50 (1.30, 1.73)

Q3 vs Q1

Q4 vs Q1

*P* for trend

0.004

<0.001

<0.001

0.573

HDP

Primary analysis

Q2 vs Q1

Q3 vs Q1

Q4 vs Q1

*P* for trend

1.12 (0.76, 1.64)

1.31 (0.90, 1.90)

1.89 (1.33, 2.67)

0.156

<0.001

<0.001

0.571

Excluding the lowest and highest 1% Q2 vs Q1

1.12 (0.76, 1.65)

1.30 (0.89, 1.90)

1.81 (1.27, 2.58)

Q3 vs Q1

Q4 vs Q1

*P* for trend

0.177

0.001

<0.001

<0.001

<0.001

<0.001

<0.001

Part B. UA as a continuous variable GDM

HDP

Primary analysis

Excluding the lowest and highest 1% Per 10 μmol/L increase 1.03 (1.02, 1.04)

Primary analysis Per 10 μmol/L increase 1.04 (1.02, 1.06)

Excluding the lowest and highest 1% Per 10 μmol/L increase 1.04 (1.02, 1.07)

Per 10 μmol/L increase 1.03 (1.02, 1.04)

**Abbreviations:** GDM, gestational diabetes mellitus; HDP, hypertensive disorders of pregnancy; RR, relative risk. Adjusted models included

hospital, gestational age at uric acid measurement, maternal age, prepregnancy body mass index, urban residence, gravidity, parity, pregestational

diabetes, chronic hypertension, thyroid disease, polycystic ovary syndrome, and kidney disease.

eTable 7.Additional Sensitivity Analyses for the Associations of Maternal Uric Acid With GDM and HDP

**Analysis**

**Outcome**

**N**

**Exposure**

**RR (95% CI)**

***P* value**

Primary cohort

GDM

5614

Q2 vs Q1

1.05 (0.90 to 1.22)

1.27 (1.10 to 1.48)

1.54 (1.34 to 1.77)

0.560

Q3 vs Q1

0.002

Q4 vs Q1

<0.001

<0.001

<0.001

0.704

*P* for trend

UA per 10 μmol/L increase

Q2 vs Q1

1.03 (1.02 to 1.04)

1.03 (0.88 to 1.21)

1.28 (1.10 to 1.48)

1.53 (1.33 to 1.76)

Excluding multiple pregnancies

GDM

5544

5574

5614

5544

Q3 vs Q1

0.001

Q4 vs Q1

<0.001

<0.001

<0.001

0.491

*P* for trend

UA per 10 μmol/L increase

Q2 vs Q1

1.03 (1.02 to 1.04)

1.06 (0.90 to 1.23)

1.29 (1.11 to 1.49)

1.55 (1.35 to 1.78)

Excluding pregestational diabetes GDM

Q3 vs Q1

<0.001

<0.001

<0.001

<0.001

0.573

Q4 vs Q1

*P* for trend

UA per 10 μmol/L increase

Q2 vs Q1

1.03 (1.02 to 1.04)

1.12 (0.76 to 1.64)

1.31 (0.90 to 1.90)

1.89 (1.33 to 2.67)

Primary cohort

HDP

HDP

Q3 vs Q1

0.156

Q4 vs Q1

<0.001

<0.001

<0.001

0.590

*P* for trend

UA per 10 μmol/L increase

Q2 vs Q1

1.04 (1.02 to 1.06)

1.11 (0.75 to 1.64)

1.31 (0.90 to 1.91)

1.89 (1.33 to 2.69)

Excluding multiple pregnancies

Q3 vs Q1

0.162

Q4 vs Q1

<0.001

<0.001

*P* for trend

UA per 10 μmol/L increase

1.04 (1.02 to 1.06)

<0.001

**Note**：RRs and 95% CIs were estimated using modified Poisson regression models with robust variance. The primary cohort included 5614

pregnancies. In the sensitivity analysis, 70 pregnancies identified as multiple gestations from hospital diagnosis records were excluded, leaving

5544 pregnancies. Uric acid quartile cut points were kept the same as those used in the primary analysis. All models were adjusted for hospital,

gestational age at uric acid measurement, maternal age, prepregnancy body mass index, urban residence, gravidity, parity, pregestational diabetes,

chronic hypertension, thyroid disease, polycystic ovary syndrome, and kidney disease. GDM indicates gestational diabetes mellitus; HDP,

hypertensive disorders of pregnancy; RR, relative risk; CI, confidence interval; UA, uric acid. In the sensitivity analysis excluding pregnancies

with pregestational diabetes, 40 pregnancies were excluded, leaving 5574 pregnancies. Because pregestational diabetes was no longer variable in

this restricted cohort, it was not included as an adjustment covariate in this sensitivity model.

eTable 8.Hospital Specific Distribution of Maternal Uric Acid and Outcome Frequencies in the Primary Analytic Cohort

**Hospital**

**N**

**Uric acid, mean (SD), μmol/L Uric acid, median [IQR], μmol/L GDM, No. (%)**

**HDP, No. (%)**

501/1741 (28.8%) 62/1741 (3.6%)

525/2548 (20.6%) 173/2548 (6.8%)

161/1325 (12.2%) 40/1325 (3.0%)

The People's Hospital of Lincang, Yunnan

1741 210.0 (62.3)

201.0 [168.0, 240.0]

222.0 [192.0, 257.0]

219.0 [192.0, 248.0]

Wuhan Maternal and Child Health Hospital 2548 228.1 (52.9)

Shanghai Tenth People's Hospital 1325 222.7 (44.7)

**Abbreviations:** GDM, gestational diabetes mellitus; HDP, hypertensive disorders of pregnancy. Uric acid values are presented as mean (SD) and

median [IQR] to describe center specific exposure distributions.

eTable 9.Hospital Stratified Associations of Maternal Uric Acid With Gestational Diabetes Mellitus and Hypertensive Disorders of Pregnancy

**Outcome**

**Hospital**

Lincang

MCH

**Events/N (%)**

**Adjusted RR (95% CI)**

1.03 (1.02, 1.04)

1.02 (1.01, 1.04)

1.05 (1.02, 1.08)

1.03 (0.98, 1.07)

1.03 (1.01, 1.06)

1.08 (1.02, 1.15)

***P* value**

<0.001

<0.001

<0.001

0.213

***P* for interaction**

GDM

501/1741 (28.8%)

525/2548 (20.6%)

161/1325 (12.2%)

62/1741 (3.6%)

173/2548 (6.8%)

40/1325 (3.0%)

0.288

Tenth

HDP

Lincang

MCH

0.225

0.009

Tenth

0.009

**Abbreviations**: GDM, gestational diabetes mellitus; HDP, hypertensive disorders of pregnancy; RR, relative risk; MCH, Wuhan Maternal and

Child Health Hospital.

**Note:** Adjusted models were fitted separately within each hospital and included gestational age at uric acid measurement, maternal age,

prepregnancy body mass index, urban residence, gravidity, parity, pregestational diabetes, chronic hypertension, thyroid disease, polycystic ovary

syndrome, and kidney disease. P for interaction was derived from pooled models including a multiplicative interaction term between maternal uric

acid and hospital. Continuous associations were expressed per 10 μmol/L increase in uric acid.

eTable 10.Leave One Hospital Out Analyses for the Associations of Maternal Uric Acid With Gestational Diabetes Mellitus and Hypertensive

Disorders of Pregnancy

**Part**

**Outcome**

**Analysis**

**Exposure**

Q2 vs Q1

Q3 vs Q1

Q4 vs Q1

P for trend

Q2 vs Q1

Q3 vs Q1

Q4 vs Q1

P for trend

Q2 vs Q1

Q3 vs Q1

Q4 vs Q1

P for trend

Q2 vs Q1

Q3 vs Q1

Q4 vs Q1

P for trend

Q2 vs Q1

Q3 vs Q1

Q4 vs Q1

P for trend

Q2 vs Q1

Q3 vs Q1

Q4 vs Q1

**Adjusted RR (95% CI) *P* value**

Part A. UA quartiles

GDM

Excluding Lincang

1.04 (0.84, 1.28)

1.41 (1.16, 1.71)

1.51 (1.25, 1.83)

0.74

<0.001

<0.001

<0.001

0.353

0.007

<0.001

<0.001

0.418

0.007

<0.001

<0.001

0.885

0.177

0.004

0.001

0.789

0.456

0.008

0.005

0.626

0.229

0.004

Excluding MCH

Excluding Tenth

Excluding Lincang

Excluding MCH

Excluding Tenth

1.10 (0.90, 1.35)

1.32 (1.08, 1.62)

1.70 (1.41, 2.04)

1.07 (0.91, 1.26)

1.25 (1.06, 1.46)

1.52 (1.30, 1.77)

Part A. UA quartiles

HDP

0.97 (0.64, 1.47)

1.31 (0.89, 1.93)

1.70 (1.18, 2.46)

1.09 (0.57, 2.09)

1.28 (0.67, 2.43)

2.15 (1.22, 3.80)

1.11 (0.73, 1.68)

1.28 (0.86, 1.92)

1.77 (1.20, 2.60)

eTable10.Leave One Hospital Out Analyses for the Associations of Maternal Uric Acid With Gestational Diabetes Mellitus and Hypertensive

Disorders of Pregnancy

**Part**

**Outcome**

**Analysis**

**Exposure**

**Adjusted RR (95% CI) *P* value**

P for trend

0.002

Part B. UA as a continuous variable

GDM

Excluding Lincang

Excluding MCH

Excluding Tenth

Excluding Lincang

Excluding MCH

Excluding Tenth

Per 10 μmol/L increase

Per 10 μmol/L increase

Per 10 μmol/L increase

Per 10 μmol/L increase

Per 10 μmol/L increase

Per 10 μmol/L increase

1.03 (1.02, 1.04)

1.03 (1.02, 1.04)

1.03 (1.02, 1.03)

1.04 (1.02, 1.07)

1.05 (1.02, 1.08)

1.04 (1.02, 1.06)

<0.001

<0.001

<0.001

<0.001

0.003

Part B. UA as a continuous variable

HDP

<0.001

**Abbreviations:** GDM, gestational diabetes mellitus; HDP, hypertensive disorders of pregnancy; RR, relative risk.

Note: Leave one hospital out analyses were performed by repeating the primary adjusted models after excluding each hospital in turn. Adjusted

models included hospital, gestational age at uric acid measurement, maternal age, prepregnancy body mass index, urban residence, gravidity, parity,

pregestational diabetes, chronic hypertension, thyroid disease, polycystic ovary syndrome, and kidney disease

eTable11.Baseline Characteristics by Ethnicity in the Subcohort With Available Ethnicity Data

**Variable**

**Han**

**Yi**

**Dai**

**Lahu**

**Wa**

**(n=74)**

**Other ethnic**

**(n=147)**

**(n=1099)**

209.5 ± 61.9

10.9 ± 3.2

30.4 ± 4.6

22.3 ± 3.5

**(n=216)**

214.8 ± 63.9

10.7 ± 3.0

30.5 ± 4.1

22.4 ± 3.3

**(n=120)**

**(n=85)**

Serum uric acid, μmol/L

208.2 ± 55.2

10.9 ± 3.9

30.8 ± 4.7

22.5 ± 3.3

208.5 ± 74.9

10.6 ± 3.0

30.9 ± 4.8

22.7 ± 3.6

203.6 ± 48.9

11.1 ± 3.3

31.5 ± 4.8

23.4 ± 3.7

213.1 ± 66.8

10.8 ± 2.9

30.4 ± 3.9

22.7 ± 3.6

Gestational age at sampling, weeks

Maternal age, years

Prepregnancy BMI, kg/m²

Categorical variables, No.

(%)

Urban residence, No. (%)

Gestational diabetes mellitus, No. (%)

Hypertensive disorders of pregnancy, No. (%)

Pregestational diabetes, No. (%)

Chronic hypertension, No. (%)

Thyroid disease, No. (%)

905 (82.3)

326 (29.7)

39 (3.5)

184 (85.2)

50 (23.1)

4 (1.9)

101 (84.2)

43 (35.8)

7 (5.8)

69 (81.2)

22 (25.9)

2 (2.4)

50 (67.6)

19 (25.7)

5 (6.8)

134 (91.2)

41 (27.9)

5 (3.4)

23 (2.1)

3 (1.4)

3 (2.5)

4 (4.7)

1 (1.4)

2 (1.4)

13 (1.2)

2 (0.9)

1 (0.8)

2 (2.4)

1 (1.4)

2 (1.4)

215 (19.6)

42 (19.4)

20 (16.7)

15 (17.6)

14 (18.9)

31 (21.1)

**Abbreviations:** BMI, body mass index.

**Note:** Data are presented as mean ± SD for continuous variables and No. (%) for categorical variables. Other ethnic groups include ethnic groups

other than Han, Yi, Dai, Lahu, and Wa.

eTable12.Associations of Maternal Uric Acid With Gestational Diabetes Mellitus and Hypertensive Disorders of Pregnancy After Additional

Adjustment for Ethnicity in the Subcohort With Available Ethnicity Data

**Outcome**

**Predictor**

**RR**

**95% CI**

***P* value**

GDM

UA (per 10 μmol/L increase)

1.028

(1.017, 1.039)

<0.001

Ethnicity (Ref: Han)

Yi

0.769

1.21

(0.598, 0.988)

(0.956, 1.530)

(0.580, 1.152)

(0.529, 1.116)

(0.693, 1.175)

(1.041, 1.080)

(1.038, 1.081)

(0.988, 1.075)

0.04

Dai

0.112

0.25

Lahu

0.818

0.768

0.902

1.06

Wa

0.166

0.446

<0.001

<0.001

0.167

Other ethnic

Age (per year)

Pre-pregnancy BMI

UA (per 10 μmol/L increase)

Ethnicity (Ref: Han)

Yi

1.059

1.03

HDP

0.521

1.734

0.615

1.88

(0.186, 1.458)

(0.821, 3.663)

(0.183, 2.066)

(0.756, 4.673)

(0.362, 2.158)

(1.026, 1.143)

(1.043, 1.190)

0.215

0.149

0.432

0.174

0.786

0.004

0.001

Dai

Lahu

Wa

Other ethnic

Age (per year)

Pre-pregnancy BMI

0.884

1.083

1.114

**Abbreviations:** BMI, body mass index; GDM, gestational diabetes mellitus; HDP, hypertensive disorders of pregnancy; RR, relative risk.

**Note:** Relative risks were estimated using modified Poisson regression with robust variance. Models were additionally adjusted for ethnicity in

the subcohort with available ethnicity data.

eTable 13.Exploratory Interaction Between Maternal Uric Acid and Ethnicity on Gestational Diabetes Mellitus and Hypertensive Disorders of

Pregnancy in the Subcohort With Available Ethnicity Data

**Outcome**

**Interaction Term (UA10** × **Ethnicity)**

Global Interaction

UA10 × Yi

***P* value (Interaction)**

0.998

GDM

0.63

UA10 × Dai

0.44

UA10 × Lahu

UA10 × Wa

0.43

0.925

UA10 × Bai

0.647

UA10 × Bulang

UA10 × Other

Global Interaction

UA10 × Yi

0.964

0.809

HDP

0.187

0.641

UA10 × Dai

0.148

UA10 × Lahu

UA10 × Wa

0.003

0.506

UA10 × Bai

0.272

UA10 × Bulang

UA10 × Other

<0.001

0.868

**Abbreviations:** GDM, gestational diabetes mellitus; HDP, hypertensive disorders of pregnancy; UA10, maternal uric acid per 10 μmol/L increase.

Note: Interaction terms represent multiplicative interaction terms between UA10 and ethnicity, with Han as the reference group. Global interaction

P values were obtained by comparing models with and without the UA10 × ethnicity interaction terms. These analyses were considered exploratory

because ethnicity information was available only in a subcohort.

eTable 14.Associations of Maternal Uric Acid With GDM and HDP Before and After Adjustment for Prepregnancy BMI

**Outcome Model**

**Adjusted RR per 10 μmol/L increase in uric acid (95% CI) *P* value**

GDM

GDM

HDP

HDP

Without adjustment for prepregnancy BMI

1.035 (1.027, 1.042)

1.030 (1.022, 1.038)

1.051 (1.031, 1.071)

1.044 (1.024, 1.065)

<.001

<.001

<.001

<.001

With adjustment for prepregnancy BMI

Without adjustment for prepregnancy BMI

With adjustment for prepregnancy BMI

**Abbreviations:** GDM, gestational diabetes mellitus; HDP, hypertensive disorders of pregnancy; RR, relative risk.

Note: Models without prepregnancy BMI included hospital, gestational age at uric acid measurement, maternal age, urban residence, gravidity,

parity, pregestational diabetes, chronic hypertension, thyroid disease, polycystic ovary syndrome, and kidney disease. Models with prepregnancy

BMI additionally included prepregnancy BMI.

eTable 15.Interaction Between Maternal Uric Acid and Gestational Age at Uric Acid Measurement for GDM and HDP

**Outcome Interaction Term**

**Likelihood Ratio Test *P* Value Robust Wald *P* Value**

GDM

HDP

UA per 10 μmol/L × gestational age at UA measurement

UA per 10 μmol/L × gestational age at UA measurement

0.55

0.411

0.483

0.532

**Abbreviations**: GDM, gestational diabetes mellitus; HDP, hypertensive disorders of pregnancy; UA, uric acid.

**Note:** Interaction was assessed by adding a multiplicative interaction term between maternal uric acid per 10 μmol/L increase and mean centered

gestational age at uric acid measurement to the fully adjusted model. Likelihood ratio tests compared models with and without the interaction term.

Robust Wald P values were examined as supportive analyses.

eTable 16a.Exploratory Prediction Performance of Established Maternal Risk Factor Models With and Without Maternal Uric Acid

**Outcome Model**

**Apparent**

**AUC**

**Bootstrap**

**Optimism**

**Corrected AUC**

0.656

**Repeated 10 Fold Cross**

**Validated AUC, Mean (95%**

**Range)**

**Apparent**

**Brier**

**Repeated 10 Fold Cross**

**Validated Brier Score**

**Score**

GDM

GDM

HDP

HDP

Clinical model

0.664

0.672

0.649

0.672

0.654 (0.648 to 0.661)

0.156

0.158

0.156

0.046

0.046

Clinical model +

maternal

0.665

0.622

0.645

0.663 (0.658 to 0.670)

0.607 (0.593 to 0.617)

0.632 (0.622 to 0.642)

0.154

0.046

0.046

uric acid

Clinical model

Clinical model +

maternal

uric acid

**Abbreviations:** AUC, area under the receiver operating characteristic curve; BMI, body mass index; GDM, gestational diabetes mellitus; HDP,

hypertensive disorders of pregnancy.

**Note:** The clinical model included hospital, gestational age at uric acid measurement, maternal age, prepregnancy BMI, urban residence, gravidity,

parity, pregestational diabetes, chronic hypertension, thyroid disease, polycystic ovary syndrome, and kidney disease. The clinical model plus

maternal uric acid additionally included maternal uric acid per 10 μmol/L increase. Bootstrap optimism correction was performed with 500

bootstrap samples. Repeated 10 fold cross validation was performed with 20 repeats. AUC values reflect model discrimination. Brier scores reflect

overall prediction error, with lower values indicating better performance.

eTable 16b.Incremental Discrimination After Adding Maternal Uric Acid

| **Outcome** | **Bootstrap Optimism Corrected AUC Difference** | **Repeated 10 Fold Cross Validated AUC Difference** |
| --- | --- | --- |
| GDM | 0.008 | 0.009 |
| HDP | 0.024 | 0.025 |

**Note:** Differences were calculated as the AUC for the clinical model plus maternal uric acid minus the AUC for the clinical model. These exploratory analyses showed modest improvement in model discrimination after adding maternal uric acid, with a larger incremental gain for HDP than for GDM.

eTable 16c. Incremental Predictive Value of Maternal Serum Uric Acid Assessed Using Continuous Net Reclassification Improvement and Integrated Discrimination Improvement

| **Outcome** | **Metric** | **Estimate (95% CI)** |
| --- | --- | --- |
| GDM | Continuous NRI | 0.174 (0.112, 0.240) |
|  | IDI | 0.010 (0.005, 0.016) |
| HDP | Continuous NRI | 0.236 (0.107, 0.364) |
|  | IDI | 0.004 (0.001, 0.009) |

**Abbreviations:** CI, confidence interval; GDM, gestational diabetes mellitus; HDP, hypertensive disorders of pregnancy; IDI, integrated discrimination improvement; NRI, net reclassification improvement.

The base model included hospital, gestational age at uric acid measurement, maternal age, prepregnancy body mass index, urban residence, gravidity, parity, pregestational diabetes, chronic hypertension, thyroid disease, polycystic ovary syndrome, and kidney disease. The extended model additionally included maternal serum uric acid per 10 μmoL/L increase. Category-free continuous NRI and IDI were calculated by comparing the extended model with the base model. Point estimates were derived from the full analytic cohort, and 95% confidence intervals were estimated using 1,000 bootstrap resamples. Category-based NRI was not calculated because validated clinical risk categories for early-pregnancy prediction of GDM or HDP were unavailable.

eTable 17.Subgroup Analyses of the Associations of Maternal Serum Uric Acid With Gestational Diabetes Mellitus and Hypertensive Disorders of Pregnancy

| **Outcome** | **Subgroup** | **Level** | **N** | **Adjusted RR (95% CI)** | ***P*-value** | ***P*-interaction** |
| --- | --- | --- | --- | --- | --- | --- |
| GDM | Prepregnancy BMI | <24 | 4321 | 1.03 (1.02, 1.04) | <0.001 | 0.426 |
|  |  | ≥24 | 1293 | 1.03 (1.01, 1.04) | <0.001 |  |
|  | Maternal age | <35 | 4782 | 1.03 (1.02, 1.04) | <0.001 | 0.844 |
|  |  | ≥35 | 832 | 1.03 (1.02, 1.05) | <0.001 |  |
|  | PCOS | Yes | 80 | 1.04 (0.99, 1.09) | 0.136 | 0.892 |
|  |  | No | 5534 | 1.03 (1.02, 1.04) | <0.001 |  |
|  | Gravidity | 1 | 3533 | 1.03 (1.01, 1.04) | <0.001 | 0.158 |
|  |  | ≥2 | 2081 | 1.03 (1.02, 1.04) | <0.001 |  |
| HDP | Prepregnancy BMI | <24 | 4321 | 1.05 (1.02, 1.07) | <0.001 | 0.607 |
|  |  | ≥24 | 1293 | 1.03 (1.00, 1.07) | 0.034 |  |
|  | Maternal age | <35 | 4782 | 1.04 (1.02, 1.06) | <0.001 | 0.340 |
|  |  | ≥35 | 832 | 1.06 (1.02, 1.10) | 0.003 |  |
|  | PCOS | Yes | 80 | 1.08 (0.97, 1.20) | 0.168 | 0.532 |
|  |  | No | 5534 | 1.04 (1.02, 1.06) | <0.001 |  |
|  | Gravidity | 1 | 3533 | 1.04 (1.02, 1.07) | <0.001 | 0.359 |
|  |  | ≥2 | 2081 | 1.04 (1.01, 1.08) | 0.012 |  |

**Abbreviations:** BMI, body mass index; CI, confidence interval; GDM, gestational diabetes mellitus; HDP, hypertensive disorders of pregnancy; PCOS, polycystic ovary syndrome; RR, relative risk.RRs represent the adjusted relative risks associated with each 10 μmoL/L increase in maternal serum uric acid. Associations were estimated using modified Poisson regression with robust variance. Models were adjusted for hospital, gestational age at uric acid measurement, maternal age, prepregnancy BMI, urban residence, gravidity, parity, pregestational diabetes, chronic hypertension, thyroid disease, PCOS, and kidney disease, excluding the variable used to define the corresponding subgroup. P values for interaction were obtained from multiplicative interaction terms between maternal serum uric acid and the corresponding subgroup variable in the full analytic cohort. Subgroup analyses were exploratory.
