# Supplementary material for: Maternal serum uric acid before 24 weeks of gestation and subsequent gestational diabetes mellitus: a multicentre retrospective cohort study
Source: Front Endocrinol (Lausanne). 2026 Jul 24;17:1896745. doi: 10.3389/fendo.2026.1896745 (PMC13449704; doi:10.3389/fendo.2026.1896745)
Supplement: Supplementary file 2 [file SupplementaryFile2.docx]

eFigure 1. Proposed metabolic and vascular pathways linking elevated maternal serum uric acid with gestational diabetes mellitus and hypertensive disorders of pregnancy.

Legend: This conceptual figure summarizes potential metabolic and vascular pathways through which elevated maternal serum uric acid may be associated with gestational diabetes mellitus and hypertensive disorders of pregnancy. Higher maternal uric acid may contribute to oxidative stress and low-grade inflammation, which may impair insulin signaling, increase peripheral insulin resistance, and increase the compensatory demand on pancreatic β cells. Possible involvement of mTOR related nutrient sensing, including interactions among mTORC1, PDX1, AMPK, and insulin or IGF 1 signaling, may further influence pancreatic β cell adaptation. Reduced nitric oxide bioavailability and endothelial dysfunction may impair maternal vascular adaptation and increase susceptibility to hypertensive disorders of pregnancy. These pathways may reflect a broader background of maternal cardiometabolic vulnerability relevant to both outcomes. The proposed mechanisms were not directly measured or tested in the present observational study. Dashed arrows indicate proposed rather than demonstrated biological relationships.

AMPK, AMP activated protein kinase; GDM, gestational diabetes mellitus; HDP, hypertensive disorders of pregnancy; IGF 1, insulin like growth factor 1; mTOR, mechanistic target of rapamycin; mTORC1, mechanistic target of rapamycin complex 1; NO, nitric oxide; PDX1, pancreatic and duodenal homeobox 1; UA, uric acid.
